# Aryl Hydrocarbon Receptor Activation by TCDD Modulates Expression of Extracellular Matrix Remodeling Genes during Experimental Liver Fibrosis

**DOI:** 10.1155/2016/5309328

**Published:** 2016-09-08

**Authors:** Cheri L. Lamb, Giovan N. Cholico, Daniel E. Perkins, Michael T. Fewkes, Julia Thom Oxford, Trevor J. Lujan, Erica E. Morrill, Kristen A. Mitchell

**Affiliations:** ^1^Biomolecular Sciences Ph.D. Program, Boise State University, Boise, ID 83725, USA; ^2^Department of Biological Sciences, Boise State University, Boise, ID 83725, USA; ^3^Biomolecular Research Center, Boise State University, Boise, ID 83725, USA; ^4^Department of Mechanical and Biomedical Engineering, Boise State University, Boise, ID 83725, USA

## Abstract

The aryl hydrocarbon receptor (AhR) is a soluble, ligand-activated transcription factor that mediates the toxicity of 2,3,7,8-tetrachlorodibenzo-*p*-dioxin (TCDD). Increasing evidence implicates the AhR in regulating extracellular matrix (ECM) homeostasis. We recently reported that TCDD increased necroinflammation and myofibroblast activation during liver injury elicited by carbon tetrachloride (CCl_4_). However, TCDD did not increase collagen deposition or exacerbate fibrosis in CCl_4_-treated mice, which raises the possibility that TCDD may enhance ECM turnover. The goal of this study was to determine how TCDD impacts ECM remodeling gene expression in the liver. Male C57BL/6 mice were treated for 8 weeks with 0.5 mL/kg CCl_4_, and TCDD (20 *μ*g/kg) was administered during the last two weeks. Results indicate that TCDD increased mRNA levels of procollagen types I, III, IV, and VI and the collagen processing molecules HSP47 and lysyl oxidase. TCDD also increased gelatinase activity and mRNA levels of matrix metalloproteinase- (MMP-) 3, MMP-8, MMP-9, and MMP-13. Furthermore, TCDD modulated expression of genes in the plasminogen activator/plasmin system, which regulates MMP activation, and it also increased TIMP1 gene expression. These findings support the notion that AhR activation by TCDD dysregulates ECM remodeling gene expression and may facilitate ECM metabolism despite increased liver injury.

## 1. Introduction

The aryl hydrocarbon receptor (AhR) is a soluble protein in the basic helix-loop-helix Per/ARNT/Sim family of transcriptional regulators that contribute to developmental processes, adaptation to environmental stress, and xenobiotic metabolism [1–3]. The AhR mediates the toxicity associated with exposure to 2,3,7,8-tetrachlorodibenzo-*p*-dioxin (TCDD), which is an environmental contaminant and high-affinity ligand for this receptor [4]. After ligand binding, the AhR translocates from the cytoplasm to the nucleus, where it forms a heterodimer with the AhR nuclear translocator protein (ARNT). The AhR/ARNT complex binds to DNA at xenobiotic response elements (XREs) and modulates gene transcription. A growing body of evidence indicates that the AhR also interacts with other coregulatory proteins in addition to ARNT and can modulate the expression of genes that do not contain XREs [5], which underscores the increasing complexity of AhR-mediated gene regulation. Such AhR-dependent changes in gene expression are believed to underlie most of the toxic responses to TCDD. In the absence of TCDD, endogenous AhR activation is implicated in regulating the expression of genes important for a number of developmental and physiological processes [6, 7].

Emerging evidence implicates a role for AhR signaling in the deposition and metabolism of extracellular matrix (ECM) components. The ECM is comprised of a network of proteins, such as collagens, which are deposited in interstitial spaces and provide mechanical and structural support to cells [8]. The ECM also regulates various cellular processes, such as survival, migration, proliferation, and differentiation, by modulating tissue stiffness, communicating with the intracellular cytoskeleton, and sequestering and releasing growth factors [9]. AhR activation by TCDD has been shown to modulate the expression of ECM proteins, such as collagen and fibronectin [10–14]. Expression of matrix metalloproteinases (MMPs), which are responsible for the degradation of ECM components, also appears to be targeted by TCDD. For example,* in vitro* TCDD treatment was found to increase MMP expression in human keratinocytes, prostate cancer cells, and melanoma cells [15–17]. Insight into the effect of TCDD on ECM maintenance and remodeling also stems from studies in a zebrafish regeneration model, in which amputation of the caudal (tail) fin initiates epimorphic regeneration accompanied by a wound healing response. Using this model, Andreasen et al. reported that TCDD treatment increased the expression of MMP-9 and MMP-13 [18]. In addition, exposure to TCDD induced a localized fibrosis in the regenerating fin, where collagen accumulated as an unorganized fibrotic deposit at the basement membrane. In a separate study, gene expression analysis revealed that the largest numbers of genes impacted by TCDD during fin regeneration were those involved in ECM remodeling and structure [10]. Collectively, these reports support the notion that TCDD dysregulates ECM homeostasis, and this most likely occurs through a mechanism that includes AhR-mediated changes in gene expression.

Disruptions of ECM metabolism and deposition are known to impact the development of liver disease [19, 20]. Liver fibrosis is a pathological condition characterized by the deposition of excessive or abnormal ECM components, including collagen type I [19]. In the liver, collagen is synthesized by myofibroblast precursors, namely, hepatic stellate cells (HSCs). Upon liver injury, HSCs transition from quiescent, vitamin A-rich cells into activated myofibroblasts, characterized by increased proliferation, contractility, and synthesis of collagen type I [21]. One well-established model system to investigate HSC activation and ECM modulation is experimental liver fibrosis induced by chronic carbon tetrachloride (CCl_4_) administration. In the liver, CCl_4_ is metabolized by cytochrome P4502E1 to a trichloromethyl radical that elicits membrane damage through lipid peroxidation [22]. Chronic treatment of mice with CCl_4_ causes widespread centrilobular necrosis and inflammation, which drive HSC activation and the development of fibrosis [23].

We recently found that exposure to TCDD increased liver damage and HSC activation in mice treated with CCl_4_ for 8 weeks [24]. However, TCDD did not increase the deposition of collagen or the severity of liver fibrosis in CCl_4_-treated mice, despite increased expression of genes encoding collagen type I and the potent profibrogenic mediator, transforming growth factor-*β*1 (TGF-*β*1). Results further indicated that TCDD increased collagenase activity in the liver of CCl_4_-treated mice. Increased breakdown of ECM in CCl_4_/TCDD-treated mice could explain why collagen deposition and fibrosis development were not exacerbated, despite increases in other endpoints of fibrogenesis.

Collagen biosynthesis begins with the transcription of procollagen genes and is facilitated by various intercellular and extracellular molecules [25]. For example, heat shock protein-47 (HSP47) is required for proper triple helical folding and trafficking of procollagen within the endoplasmic reticulum [26]. Another molecule, decorin, regulates collagen fibrillogenesis [27–29]. Lysyl oxidase (LOX) catalyzes cross-linking of collagen fibers, which marks the last step in collagen biosynthesis [30].

Collagen breakdown is achieved through the activity of numerous MMPs. MMP expression is regulated at the transcriptional level, and these proteins are synthesized as inactive zymogens called proMMPs [31]. MMP activity is regulated by enzymatic inhibition and activation. For example, endogenous tissue inhibitors of metalloproteinases (TIMPs) inhibit MMP activity. Numerous mechanisms activate MMPs, including the plasminogen activator/plasmin system [20]. Plasmin is produced through the cleavage of plasminogen by tissue plasminogen activator (tPA) and urokinase plasminogen activator (uPA), and this pathway is suppressed by plasminogen activator inhibitor-1 (PAI-1). Plasmin can directly convert proMMPs into enzymatically active MMPs, and some of these active MMPs can further activate other proMMPs [32]. MMP activity is central to the resolution of fibrosis, and scar-associated macrophages have been identified as an abundant cellular source of these enzymes in the fibrotic liver [33, 34].

The goal of the present study was to determine how TCDD treatment impacts the expression of genes related to ECM synthesis, deposition, and breakdown during chronic liver injury induced by CCl_4_ administration. We measured gene expression related to collagen synthesis, processing, and cross-linking and assessed the impact of TCDD on the organization and dispersion of fibrillar collagens in the injured liver. Expression of MMPs and the molecules that activate or inhibit them were also measured to determine how TCDD modulates ECM turnover.

## 2. Materials and Methods

### 2.1. Animals

Male C57BL/6 mice (8–10 weeks old; Charles River, Wilmington, MA) were injected i.p. with 0.5 mL/kg CCl_4_ (Sigma-Aldrich, St. Louis, MO) diluted in corn oil or with corn oil alone (Ctrl) twice a week for 8 weeks. During the last two weeks of the experiment, mice were treated by oral gavage once weekly with 20 *μ*g/kg TCDD (Cambridge Isotope Laboratories, Andover, MA) diluted in peanut oil or with peanut oil vehicle alone (Veh). At the end of the experiment, animals were euthanized, and liver was either flash-frozen in liquid nitrogen or fixed in UltraLight Zinc Formalin Fixative (PSL Equipment, Vista, CA). All animal experiments were approved by the Institutional Animal Care and Use Committee at Boise State University and conducted according to the established policies and guidelines of this committee.

### 2.2. Quantitative Real-Time RT-PCR

Total RNA was extracted using the Omega Bio-Tek E.Z.N.A.® Total RNA Kit (Norcross, GA) from 20 mg of frozen liver tissue. Genomic DNA was eliminated using the Omega RNase Free DNase Set (Norcross, GA). RNA concentration and purity were measured by ultraviolet (UV) absorbance. RNA quality and elimination of genomic DNA were assessed using an agarose bleach gel [36]. RNA was reverse-transcribed using the Applied Biosystems High Capacity cDNA reverse transcription kit (Thermo Fisher Scientific, Waltham, MA). Gene-specific primers (Table 1) were used for quantitative real-time RT-PCR (qRT-PCR), which was performed using a Light Cycler® 96 Thermocycler and FastStart*™* Essential DNA Green Master Reaction Mix (Roche, Indianapolis, IN). All samples were analyzed in duplicate from three mice per treatment group. Relative quantification was estimated using ΔΔ*C*_*q*_ method normalized to GAPDH [37].

### 2.3. Measurement of Collagen Fibril Organization

Fixed liver tissue was paraffin-embedded, cut into 2 *μ*m sections, and stained with Sirius Red as described elsewhere [38]. Birefringence of stained liver tissues was visualized using an Olympus BX53F polarizing microscope. Photographs were taken at 600x magnification to focus on septa formation in the damaged liver of CCl_4_-treated mice. Images were then converted to 8 bit grayscale and analyzed with FiberFit software to calculate fiber dispersion (*κ*) [35]. Ten images were analyzed from each mouse liver; four mice were evaluated per treatment group. Septa formation was not detected in the livers of vehicle- or TCDD-treated mice that did not receive CCl_4_, and these samples were excluded from the FiberFit analysis.

### 2.4. *In Situ* Zymography

Gelatinase activity was examined using* in situ* zymography of zinc-formalin-fixed liver tissue as described elsewhere [39, 40]. Briefly, tissue sections (8 *μ*m) were heated at 58°C for 12 hours then deparaffinized and rehydrated. DQ*™*-gelatin (Thermo Fisher Scientific) was dissolved in reagent-grade water and diluted at 1 : 50 in a 50 mM Tris-HCl buffer containing 150 mM NaCl and 5 mM CaCl_2_ (pH 7.6). Tissue sections were incubated with the DQ*™*-gelatin solution for 12 hours at 37°C. Nuclei were stained with 4′,6-diamidino-2-phenylindole (DAPI), and cover slips were mounted with Permount (Fisher Scientific, Pittsburgh, PA). Fluorescent images were taken with an EVOS*™* fluorescence microscope (Thermo Fisher Scientific) with 20x objective. Fluorescence was quantified using ImageJ software (US National Institutes of Health) and expressed as a percentage of the area in the microscope field of view.

### 2.5. Western Blotting

Frozen liver tissue was homogenized in 50 mM HEPES, 150 mM NaCl, 10% glycerol, 0.1% Tween 20, 7.5 mM EDTA, and 7.5 mM MgCl_2_ *∗* 6H_2_O. Protein content was determined using a* DC™* Protein Assay kit (Bio-Rad Laboratories, Inc., Hercules, CA), and homogenates were diluted to 5 mg/mL and resuspended in SDS loading buffer (100 mM Tris-Cl pH 6.8, 4% SDS, 0.2% bromophenol blue, and 20% glycerol) containing 400 mM *β*-mercaptoethanol. Samples (25 *μ*g protein/lane) were resolved on a 10% SDS-polyacrylamide gel, transferred to nitrocellulose, and incubated with the following antibodies purchased from Santa Cruz Biotech (Dallas, TX): anti-actin (sc-1615), anti-uPa (sc-59727), or anti-tPA (sc-5239). Blots were then incubated with HRP-conjugated secondary antibodies, and bands were visualized with Pierce*™* ECL Western Blotting Substrate (Thermo Scientific).

### 2.6. Immunohistochemistry

Liver tissue was fixed in UltraLight Zinc Formalin Fixative (PSL Equipment, Vista, CA), paraffin-embedded, and cut into 2 *μ*m sections. Tissue sections were incubated overnight at 4°C with an anti-F4/80 antibody (#MCA497R, AbD Serotec, Raleigh, NC) and then stained with 3,3-diaminobenzidine (DAB) using a commercially available kit (R&D Systems, Minneapolis, MN). Tissues were counterstained with hematoxylin. Images were taken with an Olympus BX53 compound microscope at 10x and 20x magnifications.

### 2.7. Statistical Analysis

Statistical analyses were performed using Prism (version 6; GraphPad Software, La Jolla, Ca.). With the exception of data in Figure 3, all data were evaluated by two-way analysis of variance followed by Bonferroni's* post hoc* testing to evaluate significance among all possible pairwise comparisons in the four treatment groups. Statistical significance between pairwise comparisons is indicated with letters above the bar for each treatment group. If two groups share the same letter, then the difference between the means is not statistically significant at *p* < 0.05. If two means have different letters, then they are significantly different from each other at *p* < 0.05. For the analysis of collagen fiber organization in Figure 3, an unpaired, two-tailed Student's *t*-test was used, and data were also considered significantly different at *p* < 0.05.

## 3. Results

### 3.1. Consequences of TCDD Treatment on Procollagen mRNA Levels in CCl_4_-Treated Mice

**Figure 1:**
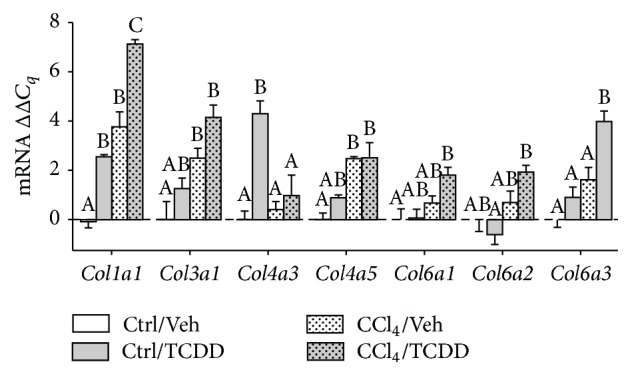
Consequences of TCDD treatment on collagen mRNA levels in the liver of CCl_4_-treated mice. Collagen mRNA expression was measured by qRT-PCR and normalized to GAPDH. Data represent mean (±SEM) of three mice per treatment group. Within the data set for each gene, all possible pairwise comparisons were measured. Means that do not share a letter are significantly different from each other (*p* < 0.05), whereas means that share a letter are not.

To determine how TCDD treatment impacts procollagen synthesis during chronic liver injury, we measured the mRNA levels of genes that encode procollagen type I and III (fibrillar collagens) and types IV and VI (nonfibrillar collagens). Chronic CCl_4_ treatment significantly increased* Col1a1*,* Col3a1*, and* Col4a5* in the mouse liver (Figure 1). Administration of TCDD to CCl_4_-treated mice further increased expression of* Col1a1* compared to mice that received CCl_4_ alone. The combination of TCDD and CCl_4_ markedly increased transcript levels of* Col6a1*,* Col6a2*, and* Col6a3* compared to mice that did not receive CCl_4_. Finally, TCDD treatment elevated* Col4a3* mRNA levels in mice that were not treated with CCl_4_, but this increase was not observed in mice that received both TCDD and CCl_4_. Collectively, these findings support a general trend in which exposure to TCDD increases procollagen gene expression in the liver of CCl_4_-treated mice. Moreover, TCDD impacts the expression of procollagen isoforms that encode both fibrillar and nonfibrillar types of collagens.

### 3.2. TCDD Modulates mRNA Levels of Collagen Processing Molecules in CCl_4_-Treated Mice

**Figure 2:**
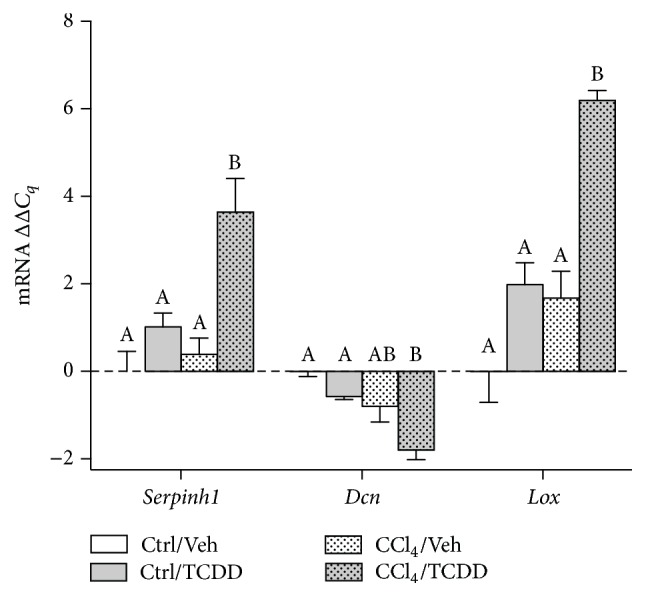
TCDD treatment alters expression of collagen processing molecules in the liver of CCl_4_-treated mice. Transcript levels of* Serpinh1* (HSP47),* Dcn* (decorin), and* Lox* (lysyl oxidase) were measured by qRT-PCR and normalized to GAPDH. Data represent mean (±SEM) of three mice per treatment group. Within the data set for each gene, all possible pairwise comparisons were measured. Means that do not share a letter are significantly different from each other (*p* < 0.05), whereas means that share a letter are not.

Collagen synthesis requires not only expression of procollagen genes but also processing of procollagen, assembly of fibrils, and cross-linking of fibers. To identify how TCDD impacts these events during CCl_4_-induced liver injury, we measured transcript levels of* Serpinh1* (HSP47),* Lox* (LOX), and* Dcn* (decorin). HSP47 is required for proper folding and trafficking of procollagen, whereas decorin and lysyl oxidase contribute to fibril assembly and fiber cross-linking in the ECM [26, 41]. When TCDD was administered to CCl_4_-treated mice,* Serpinh1* and* Lox* mRNA levels increased 4- to 6-fold compared to mice treated with CCl_4_ alone (Figure 2). In contrast,* Dcn* mRNA levels were significantly decreased in CCl_4_/TCDD-treated mice. These results support the notion that TCDD modulates the expression of genes encoding collagen-processing molecules during chronic liver injury.

### 3.3. TCDD Treatment Does Not Affect Collagen Fiber Organization in the Liver of CCl_4_-Treated Mice

**Figure 3:**
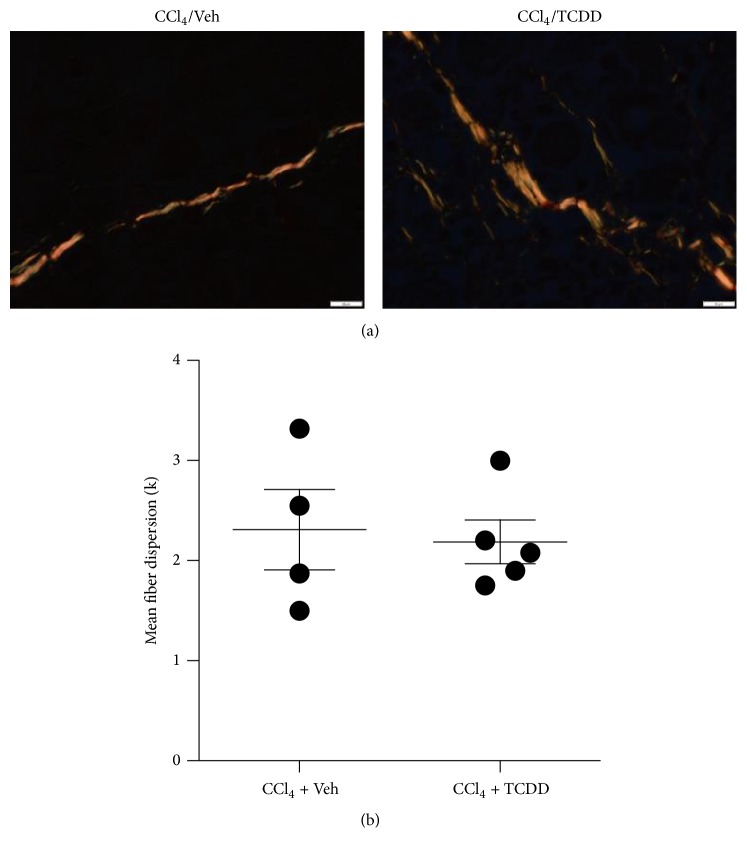
Exposure to TCDD does not impact collagen fiber organization in the liver of CCl_4_-treated mice. (a) Polarized microscopy facilitates the visualization of collagen fiber birefringence in liver tissue stained with Sirius Red (600x magnification). Photomicrographs depict representative fibers in septa of liver from a mouse treated with CCl_4_ and peanut oil vehicle (left) or with CCl_4_ and TCDD (right). Scale bars represent 10 *μ*m. (b) Collagen network organization was evaluated by analyzing Sirius Red-stained liver tissues with the FiberFit software application [35]. Ten photomicrographs were evaluated per mouse; four mice were analyzed in each treatment group. Data represent mean (±SEM) fiber dispersion, *k* (greater *k* values = increase in fiber alignment). No statistically significant changes were found between treatment groups (*p* = 0.36 based on unpaired, two-tailed Student's *t*-test).

The observation that TCDD altered the expression of ECM processing molecules in CCl_4_-treated mice led us to speculate that it would subsequently impact the fibrillar collagen network. To test this, collagen fibers were visualized in liver tissue stained with Sirius Red, which aligns with fibrillar collagens and enhances their birefringence under cross-polarized light [42, 43]. Polarized microscopy of stained tissue revealed the presence of thick, strongly birefringent yellow fibers in the septa of livers from CCl_4_-treated mice (Figure 3(a)). Based on visual inspection, TCDD had no overt impact on fiber appearance. The effects of TCDD on collagen fiber organization were further evaluated using the free software application, FiberFit, which uses image processing techniques to analyze two-dimensional images of fiber networks [35]. Results indicate that TCDD had no effect on fiber dispersion, which is a measure of fiber network disorder (Figure 3(b)). Hence, despite the TCDD-mediated increase in expression of genes encoding procollagen and collagen-processing molecules, TCDD did not appear to impact collagen organization in the ECM of CCl_4_-treated mice. No collagen fibers were detected in either vehicle- or TCDD-treated mice that did not receive CCl_4_ (data not shown), and these samples were excluded from the FiberFit analysis.

### 3.4. Expression of ECM Remodeling Enzymes Is Elevated in the Presence of TCDD

**Figure 4:**
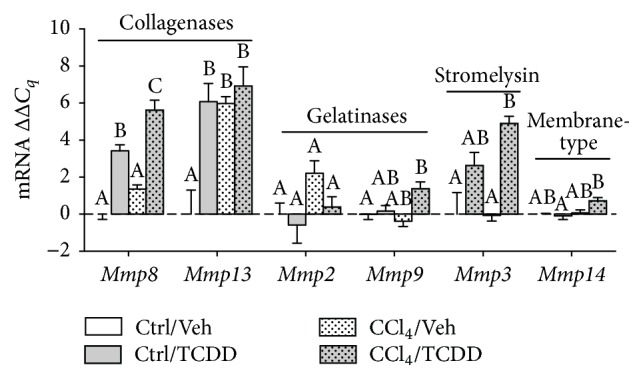
Effects of TCDD treatment on mRNA levels of MMPs in the liver of CCl_4_-treated mice. MMP mRNA expression was measured by qRT-PCR and normalized to GAPDH. Data represent mean (±SEM) of three mice per treatment group. Within the data set for each gene, all possible pairwise comparisons were measured. Means that do not share a letter are significantly different from each other (*p* < 0.05), whereas means that share a letter are not.

ECM maintenance requires not only the synthesis and deposition of matrix molecules but also their degradation and turnover, which is regulated by the proteolytic activity of MMPs. MMP expression is largely regulated at the transcriptional level [44]. To determine how TCDD treatment impacts MMP gene expression in the liver of CCl_4_-treated mice, we measured transcript levels of mouse MMPs known to be important in chronic liver injury.* Mmp8* and* Mmp13* encode enzymes that function primarily as collagenases, and expression of these genes was markedly increased by TCDD regardless of CCl_4_ treatment (Figure 4).* Mmp2* and* Mmp9* are referred to as gelatinases, and they degrade not only gelatin but also collagen type IV, laminin, elastin, and fibronectin [44]. While TCDD had no effect on* Mmp2* transcript levels, it increased* Mmp9* expression in CCl_4_-treated mice. Likewise, the combination of TCDD and CCl_4_ increased* Mmp14* (membrane-type MMP) expression compared to mice treated with TCDD alone, although this increase was modest.* Mmp3* (stromelysin) mRNA levels were significantly higher in TCDD-treated mice, regardless of CCl_4_ treatment. Generally speaking, these results support the conclusion that TCDD treatment increases MMP gene expression during CCl_4_-induced liver injury.

### 3.5. TCDD Increases Gelatinase Activity in the Liver of CCl_4_-Treated Mice

**Figure 5:**
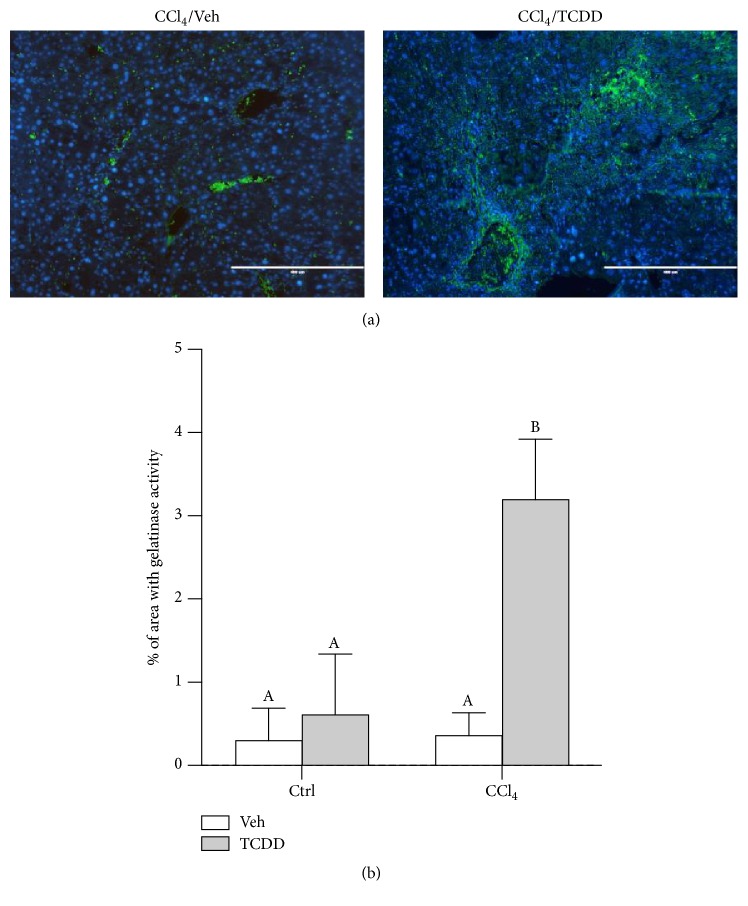
TCDD treatment increases gelatinase activity in the liver of CCl_4_-treated mice. (a)* In situ* zymography of zinc-buffered, formalin-fixed liver tissue using DQ*™*-gelatin. Green fluorescence indicates gelatinase activity; nuclei were stained with DAPI. Photomicrographs (100x magnification) are representative of three mice per treatment group. Scale bars represent 400 *μ*m. (b) Quantification of gelatinase activity based on the percentage of green fluorescence coverage per field of liver tissue. Ten fields were analyzed per mouse; three mice were evaluated per treatment group. Data represent mean (±SEM) of three mice per treatment group. All possible pairwise comparisons were measured for statistical significance. Means that do not share a letter are significantly different from each other (*p* < 0.05), whereas means that share a letter are not.

MMP activity is central to ECM remodeling and is implicated in both the promotion and attenuation of liver injury [20]. We recently found that TCDD treatment increased collagenase activity in the liver of CCl_4_-treated mice [24]. During fibrotic liver injury, collagenases cleave the native helix of fibrillar collagens to produce gelatin, which can be degraded by MMPs, namely, MMP-2 and MMP-9 [45]. We used* in situ* zymography to measure gelatinase activity in the liver. Whereas gelatinase activity was barely detectable in mice treated with CCl_4_/Veh (Figure 5(a)), it was significantly induced when TCDD was administered to CCl_4_-treated mice (Figure 5(b)). When administered alone, TCDD did not increase gelatinase activity. In fact, there was no detectable gelatinase activity in either vehicle- or TCDD-treated mice in the absence of CCl_4_ (data not shown).

### 3.6. Consequences of TCDD Treatment on TIMP mRNA Expression in CCl_4_-Treated Mice

**Figure 6:**
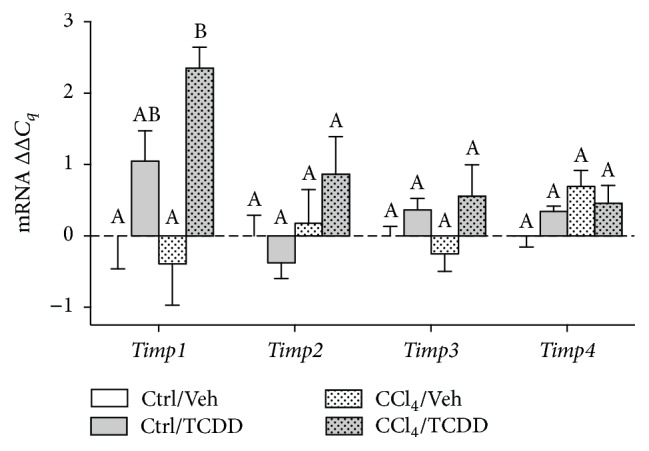
Consequences of TCDD treatment on TIMP mRNA levels in the liver of CCl_4_-treated mice. TIMP mRNA expression was measured by qRT-PCR and normalized to GAPDH. Data represent mean (±SEM) of three mice per treatment group. Within the data set for each gene, all possible pairwise comparisons were measured for statistical significance. Means that do not share a letter are significantly different from each other (*p* < 0.05), whereas means that share a letter are not.

MMP activity is controlled by enzymatic activation and inhibition [31]. The activity of MMPs can be inhibited by four homologous members of the TIMP family. TIMP1 is a strong inhibitor of many MMPs, but the gelatinases MMP-2 and MMP-9 are also inhibited by other TIMPs. For example, TIMP2, TIMP3, and TIMP4 can inhibit MMP-2 activity, and TIMP3 inhibits MMP-9 [31]. Analysis of TIMP gene expression revealed that TCDD treatment increased* Timp1* but had no impact on* Timp2*,* Timp3*, or* Timp4* regardless of CCl_4_ treatment (Figure 6). Hence, modulation of TIMP gene expression by TCDD appears to be limited to* Timp1*.

### 3.7. TCDD Treatment Modulates Expression of Molecules in the Plasminogen Activator/Plasmin System

**Figure 7:**
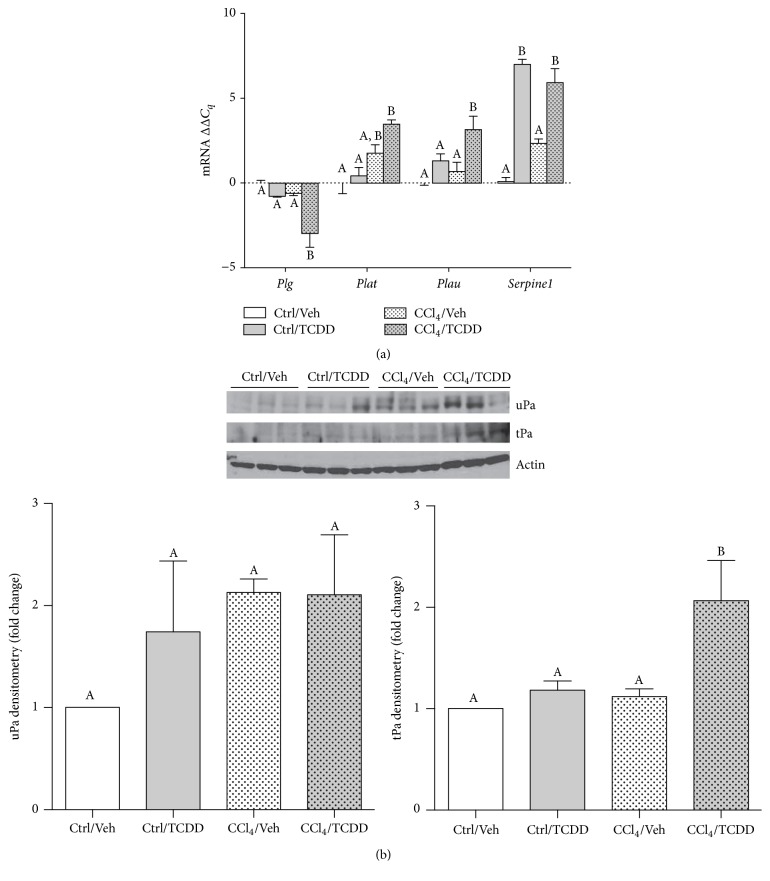
Exposure to TCDD modulates expression of genes in the plasminogen activator/plasmin system. (a) Transcript levels of* Plg* (plasminogen),* Plat* (tPA),* Plau* (uPA), and* Serpine1* (PAI-1) were measured by qRT-PCR and normalized to GAPDH. Data represent mean (±SEM) of three mice per treatment group. Within the data set for each gene, all possible pairwise comparisons were measured for statistical significance. Means that do not share a letter are significantly different from each other (*p* < 0.05), whereas means that share a letter are not. (b) uPa and tPa protein levels were measured by Western blot. Band densitometry was normalized to actin and expressed as fold change relative to the Ctrl/Veh treatment group. Means that do not share a letter are significantly different from each other (*p* < 0.05).

MMP activation is regulated through numerous mechanisms, including the plasminogen activator/plasmin system, in which tPA and uPA mediate the conversion of plasminogen to plasmin, which directly activates numerous proMMPs [20]. PAI-1 suppresses MMP proteolytic activity by inhibiting tPA/uPA, and PAI-1 gene is known to be regulated by AhR activity [46–48]. To determine how TCDD impacted this pathway of MMP activation, we measured expression of* Plg* (plasminogen),* Plat* (tPA),* Plau* (uPA), and* Serpine1* (PAI-1). TCDD induced a modest, yet statistically significant, decrease in* Plg* mRNA levels in CCl_4_-treated mice (Figure 7(a)). Levels of* Plat* and* Plau* expression were markedly elevated in CCl_4_/TCDD-treated mice. A corresponding increase in the* Plat*-encoded protein tPa was measured in CCl_4_/TCDD-treated mice, whereas no changes were detected in the expression of uPa, which is encoded by* Plau* (Figure 7(b)). Finally, exposure to TCDD increased PAI-1 (*Serpine1*) gene expression regardless of CCl_4_-treatment (Figure 7(a)). Hence, these observations indicate that TCDD treatment modulated the expression of the plasminogen activator/plasmin system.

### 3.8. TCDD Treatment Increases the Localization of Hepatic Macrophages around Fibrotic Scars in the Liver of CCl_4_-Treated Mice

**Figure 8:**
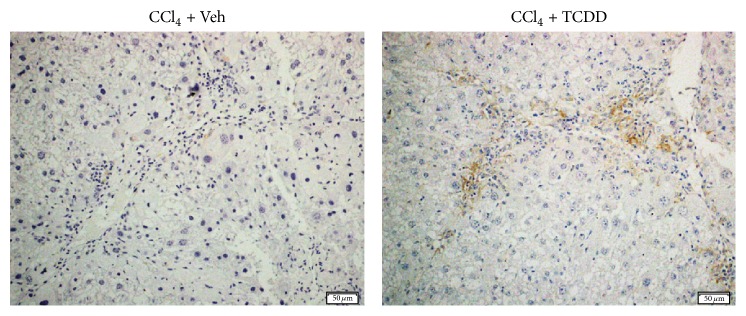
TCDD treatment increases localization of F4/80^+^ macrophages around fibrotic scar. Immunohistochemistry was performed to identify hepatic macrophages (F4/80^+^ cells) localized around the fibrotic scar in CCl_4_-treated mice (20x magnification). Scale bars represent 50 *μ*m.

Macrophages contribute to ECM remodeling during both the injury and recovery phase of CCl_4_-induced liver fibrosis [49] and are an abundant source of MMP-9 and MMP-13 [33, 50]. Scar-associated macrophages populate the fibrotic scar during injury and repair, produce MMPs, and secrete cytokines that induce MMP production in other cells [51]. Given that TCDD treatment increased MMP expression and activity, we investigated the possibility that TCDD increased the prevalence of hepatic macrophages around scar areas in the fibrotic liver. As shown in Figure 8, CCl_4_ administration elicited the infiltration of inflammatory cells to the fibrotic scar, and the prevalence of F4/80^+^ hepatic macrophages was markedly increased in CCl_4_/TCDD-treated mice compared to CCl_4_/Veh-treated mice.

## 4. Discussion

The present study investigated the consequences of TCDD treatment on expression of molecules involved in collagen biosynthesis and ECM metabolism during chronic liver injury. We recently reported that exposure to TCDD increased HSC activation and mRNA levels of TGF-*β*1 and collagen type I in the injured liver without increasing hepatic collagen content or exacerbating fibrosis [24]. This led us to speculate that TCDD treatment may dysregulate ECM remodeling activities, including collagen synthesis or turnover.

During fibrosis, the collagen content in the liver can increase up to tenfold [52]. Our results indicate that TCDD treatment alone increased* Col1a1* and* Col4a3*. This observation corroborates other reports in which exposure to TCDD increased collagen types I and IV [11–14, 53]. In CCl_4_/TCDD-treated mice, there was a marked increase in expression of* Col3a1*,* Col4a5, Col6a1*,* Col6a2*, and* Col6a3* compared to Ctrl/Veh-treated mice. Collagen type III is structurally similar to collagen type I and is the first collagen to increase during chronic liver disease [54]. Collagen type IV is the primary component of basement membranes, and its expression increases during fibrosis [55]. Collagen type VI is also upregulated in liver fibrosis and has been shown to stimulate DNA synthesis and inhibit apoptotic cell death in HSCs* in vitro* [56]. This is intriguing because we previously reported that exposure to TCDD increases HSC proliferation* in vitro* [57] and increases HSC activation markers in the liver of CCl_4_-treated mice [24]. It is possible that increased expression of collagen type VI, as well as other types of collagen, contributes to the effects of TCDD in the CCl_4_ model system.

The finding that certain collagen genes were upregulated by TCDD treatment only, while others were increased by the combination of TCDD and CCl_4_, implies that the AhR may differentially regulate gene expression in the healthy and injured liver. It is well established that most, if not all, of the biochemical and toxic effects of TCDD occur through the AhR [2, 58]. Increasing evidence supports a role for endogenous AhR signaling in regulating collagen deposition, including the discovery that AhR knockout mice develop liver fibrosis and have elevated TGF-*β*1 and collagen expression [59–61]. In addition, it was recently reported that AhR knockdown increased* Col1a1* and* Col4a4* mRNA levels in retinal pigment epithelial cells and choroidal endothelial cells [62]. Collectively, these findings implicate a role for AhR activity in regulating collagen gene expression. Future studies that investigate how AhR knockdown impacts gene expression during chronic liver injury will expand our understanding of how the AhR regulates ECM remodeling during states of health and disease. Furthermore, the use of mice in which the AhR is conditionally depleted from discrete liver cell populations could help identify which cells are directly targeted by TCDD to produce ECM dysregulation.

Not only did TCDD increase the expression of collagen genes but it also modulated gene expression for several key proteins involved in collagen synthesis. For example, administration of TCDD to CCl_4_-treated mice increased gene expression of HSP47, which resides in the endoplasmic reticulum and is involved in the folding and shuttling of collagen molecules to the Golgi [63]. Increased HSP47 levels reportedly contribute to fibrosis by facilitating the excessive assembly and intracellular processing of procollagen molecules, leading to fibrotic lesions [64]. Furthermore, suppression of HSP47 expression was reported to reduce collagen accumulation and delay fibrotic progression [65]. Both procollagen and HSP47 gene expression are regulated by TGF-*β*1 [66]. We previously found that TGF-*β*1 gene expression was increased in CCl_4_/TCDD-treated mice and speculate that this could drive HSP47 and* Col1a1* expression in our model system. However, TCDD treatment was shown to suppress both* Col1a1* and HSP47 gene expression during fin regeneration in zebrafish, despite increased TGF-*β*1 expression [10, 18].

Decorin is a secreted proteoglycan that has a dual role in liver fibrosis. First, it functions as a naturally occurring TGF-*β*1 antagonist, and its genetic ablation has been shown to increase ECM deposition, impair matrix degradation, and increase HSC activation [67]. Second, decorin facilitates the development of normal collagen morphology by binding to the collagen triple helix and preventing the lateral fusion of fibrils [28]. We found that TCDD suppressed decorin gene expression in CCl_4_-treated mice. Other studies demonstrate a possible role for AhR signaling in decorin expression. For instance, decorin expression was increased in fibroblasts and vascular smooth muscle cells from AhR knockout mice [68, 69].

LOX mediates the cross-linking of collagen fibers which is important for collagen organization and perhaps also for conferring resistance to proteolytic degradation by MMPs [70]. Consistent with this role of LOX, administration of the irreversible LOX inhibitor *β*-aminopropionitrile (BAPN) to CCl_4_-treated mice was recently found to reduce collagen cross-linking and produced fibrotic septa with less organized collagen fibers [30]. Our finding that TCDD increased LOX expression in CCl_4_-treated mice could possibly be explained as a compensatory response to increased collagen synthesis, as could the TCDD-induced increase in HSP47. It is worth noting that Andreasen et al. reported that TCDD treatment suppressed not only LOX2 and HSP47 expression during zebrafish fin regeneration but also prolyl-4-hydroxylase *α*1 and 2, which help stabilize collagen cross-links [10]. Based on the role of these molecules in collagen processing and organization, their reduced expression may underlie the accumulation of disorganized collagen observed in the regenerating fin tissue [18]. In contrast, we found no evidence that TCDD impacted collagen fiber organization in the liver of CCl_4_-treated mice. Increased expression of LOX and HSP47, as well as decreased expression of decorin, could be one possible explanation for this observation.

One of the most consistently reported consequences of TCDD treatment on ECM remodeling is increased MMP expression [71]. TCDD treatment increases the expression and activity of MMPs in numerous and diverse cell types, including keratinocytes, macrophages, and endometrial cells [72–74]. In the zebrafish model of fin regeneration, TCDD upregulated MMP-13 [10]. Similarly, TCDD increased expression of MMP-13, as well as other MMPs, in the fetal mouse heart [53]. These reports support our observation that TCDD increased* Mmp3*,* Mmp8*,* Mmp9*,* Mmp13*, and* Mmp14* genes in the mouse liver. MMP-8 and MMP-13 function primarily as collagenases, and these were robustly increased by TCDD regardless of CCl_4_ treatment, which corroborates our previous finding that TCDD increases collagenase activity in the liver of CCl_4_-treated mice [24]. During ECM breakdown, MMPs with collagenase activity will partially denature collagen, resulting in the production of gelatin, which is metabolized primarily by the gelatinases MMP-2 and MMP-9 [20]. Decreased gelatinase activity, particularly MMP-2, is associated with increased liver fibrosis development [75]. The increase in gelatinase activity we observed in CCl_4_/TCDD-treated mice could reflect a compensatory response to increased collagenase activity. Furthermore, TCDD also increased expression of MMP-3 (stromelysin) and MMP-14 (membrane-type), both of which reportedly exhibit some collagenase and gelatinase activity.

MMP activity is inhibited through interactions with TIMP proteins, as well as other endogenous inhibitors [76]. TIMP1, in particular, is associated with ECM proteolysis during fibrosis, and* Timp1* ^−/−^ mice display increased liver injury, inflammation, and fibrosis following CCl_4_ treatment [77]. TIMP1 is a strong inhibitor of most MMPs except some of the membrane-type MMPs. However, the gelatinase MMPs are inhibited by other TIMPs as well. Specifically, TIMP1 and TIMP3 inhibit MMP-9, and TIMPs 2, 3, and 4 inhibit MMP-2 [78]. In the CCl_4_ model system, TCDD treatment increased TIMP1 but had no effect on expression of TIMPs 2, 3, or 4. Thus, it is possible that the expression of TIMPs in CCl_4_/TCDD-treated mice was not sufficient to counteract MMP activity. Other studies have reported that TIMP expression is modulated by* in vitro* and* in vivo* TCDD exposure as well [10, 79–81].

Our results demonstrate that TCDD treatment produced changes in the plasminogen activator/plasmin system that modulates MMP activation. TCDD was found to modestly but significantly decrease plasminogen expression in CCl_4_-treated mice. Because MMPs are activated by plasmin, which is produced from plasminogen, this would presumably lead to decreased MMP activation. Given that TCDD increased both collagenase and gelatinase activity in the CCl_4_ model system, it is possible that the observed decrease in plasminogen gene expression was not physiologically relevant. It is also possible that increased expression of tPA and uPA compensated for any decrease in plasminogen expression. The TCDD-mediated increase in uPA gene expression corroborates another report showing that TCDD upregulated uPA protein in a human keratinocyte cell line [82]. It is interesting to note that this TCDD-induced increase in uPA appeared to occur through a posttranscriptional mechanism that included changes in mRNA stability [82, 83]. We found no significant increase in uPa protein expression among all four treatment groups in our study, although protein levels of tPa were markedly increased in CCl_4_/TCDD-treated mice.

The* Serpine1* gene that encodes PAI-1 is recognized as an AhR-regulated target gene. It is transcriptionally induced by TCDD through a mechanism that involves heterodimerization of the AhR with the transcription factor, KLF-6, and the recruitment of this complex to a nonconsensus XRE [46–48]. We found that TCDD treatment increased PAI-1 gene expression regardless of CCl_4_ treatment and presume that this reflects a direct effect of TCDD through AhR-regulated gene expression. However, it is also possible that increased PAI-1 expression by TCDD occurs as a consequences of activation of the TGF-*β*1 pathway, as PAI-1 is an early TGF-*β*1 activated gene [84], and other studies have described crosstalk between the AhR and TGF-*β*1 signaling axes [69, 85]. Based on our finding that TCDD did not suppress collagenase or gelatinase activity in CCl_4_-treated mice, it is possible that increased PAI-1 expression in CCl_4_/TCDD-treated mice failed to offset increased tPA/uPA activity. However, MMPs can also be activated through nonplasmin pathways, which leaves open the possibility that MMP activation is increased in CCl_4_/TCDD-treated mice, despite inhibition of the plasminogen activator/plasmin system by PAI-1.

The observation that TCDD treatment may enhance the prevalence of scar-associated macrophages is intriguing because these cells are a rich source of MMPs and may contribute to both the injury and regression phase of fibrosis induced by CCl_4_ [49]. We have previously found that TCDD treatment increases liver damage and inflammation in CCl_4_-treated mice [24], yet the direct cellular targets of TCDD in this model system have not been determined. It is possible that increased hepatocellular necrosis in CCl_4_/TCDD-treated mice evokes a heightened inflammatory response, resulting in increased numbers of infiltrating neutrophils and monocytes/macrophages. However, it is also conceivable that TCDD treatment directly modulates hepatic macrophages in CCl_4_-treated mice. Further investigation is warranted to identify the cellular source of increased MMP activity and determine how TCDD treatment impacts the localization of resident macrophages and infiltrating monocytes to the fibrotic scar.

**Figure 9:**
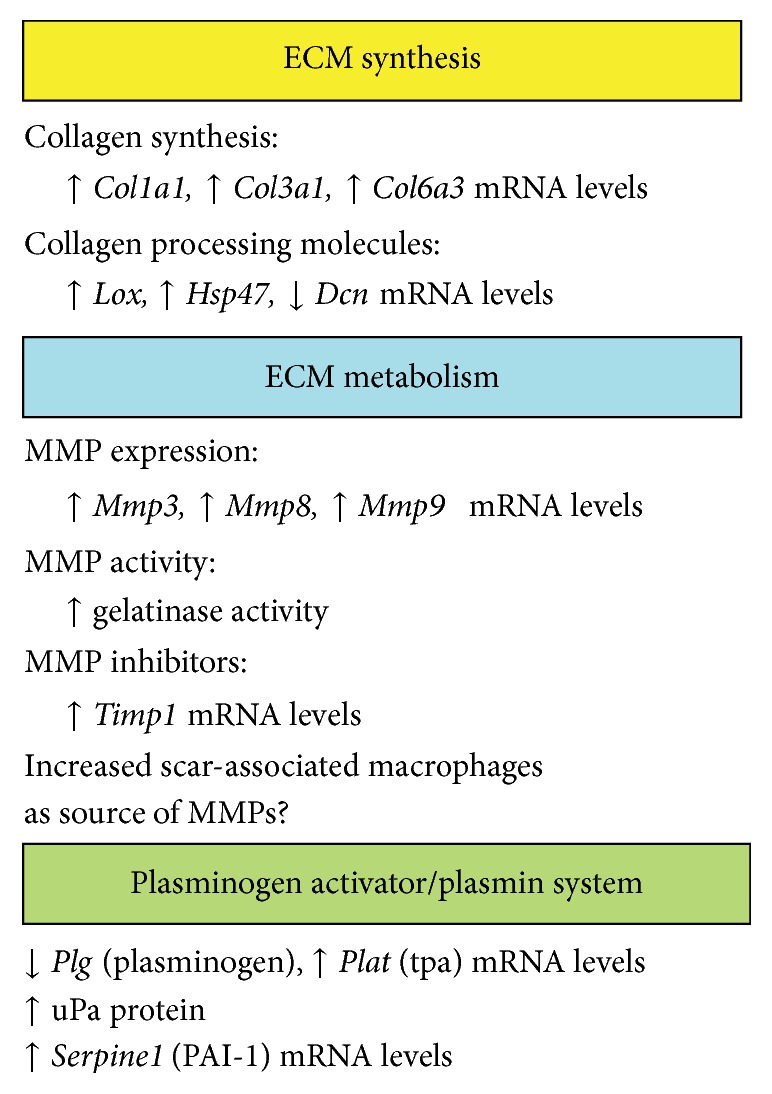
Summary of the consequences of TCDD treatment on ECM remodeling activities and regulatory processes during CCl_4_-induced liver injury.

In conclusion, results from this study demonstrate that AhR activation by TCDD modulates ECM remodeling during chronic liver injury, although the precise mechanism remains unclear. At least three interrelated components of ECM homeostasis could be targeted by TCDD: collagen synthesis, ECM metabolism, and regulation of enzyme activity by the plasminogen activator/plasmin system. As summarized in Figure 9, TCDD treatment increased expression of procollagen genes and altered expression of molecules involved in collagen processing and maturation. Furthermore, TCDD enhanced gelatinase activity, increased mRNA levels of several MMPs, and increased the localization of hepatic macrophages to the fibrotic scar. Finally, TCDD treatment had multiple effects on the plasminogen activator/plasmin system. Future studies will be needed to distinguish between TCDD-induced changes that directly impact ECM remodeling and changes that occur as secondary, compensatory responses to system perturbations.

## Figures and Tables

**Table 1:** qRT-PCR primers and annealing temperatures used in this study.

*Gene*	Primer sequence	Annealing temp. (°C)
*Col1a1*	FWD: GTCCCTGAAGTCAGCTGCATA	60
	REV: TGGGACAGTCCAGTTCTTCAT	

*Col3a1*	FWD: CCTGGTGGAAAGGGTGAAAT	62
	REV: CGTGTTCCGGGTATACCATTAG	

*Col4a3*	FWD: TCCTGGGGAAATGGGAAAGC	64
	REV: CTGCCTACGGATGGTTCTCC	

*Col4a5*	FWD: TGCTCCTGAGAGATCGGCTT	58
	REV: GTTATGCTGGTGCACTTGGG	

*Col6a1*	FWD: TCCCACCCACACAGAACAAC	58
	REV: CACTGAGAGGTGTCGTGTCC	

*Col6a2*	FWD: TGACGCTGTTCTCTGACCTG	58
	REV: TTGTGGAAGTTCTGCTCGCC	

*Col6a3*	FWD: CTGATGGCACCTCTCAGGAC	58
	REV: GTCACTTCCAACATCGAGGC	

*Dcn*	FWD: AAGGGGGCCGATAAAGTTTC	58
	REV: CTGGGTTGAAAACCTCCTGC	

*Lox*	FWD: CTGCACACACACAGGGATTG	56
	REV: AGCTGGGGTTTACACTGACC	

*Mmp2*	FWD: ACCCAGATGTGGCCAACTAC	63
	REV: TACTTTTAAGGCCCGAGCAA	

*Mmp3*	FWD: GTCCTCCACAGACTTGTCCC	65
	REV: GGGAGTTCCATAGAGGGACTGA	

*Mmp8*	FWD: TACAGGGAACCCAGCACCTA	64
	REV: GGGGTTGTCTGAAGGTCCATAG	

*Mmp9*	FWD: AAGGCAGCGTTAGCCAGAAG	63
	REV: GCGGTACAAGTATGCCTCTGC	

*Mmp13*	FWD: GCCCTGGGAAGGAGAGACTCCAGG	55
	REV: GGATTCCCGCAAGAGTCGCAGG	

*Mmp14*	FWD: GCCCTCTGTCCCAGATAAGC	58
	REV: ACCATCGCTCCTTGAAGACA	

*Plat*	FWD: CAGAGATGAGCCAACGCAGA	58
	REV: TTCGCTGCAACTTCGGACAG	

*Plau*	FWD: CATCCAGTCCTTGCGTGTCT	62
	REV: CCAAGTACACTGCCACCTTCA	

*Plg*	FWD: ACTCAAGGGACTTTCGGTGC	58
	REV: TCAGATACTCGACGCGGTTG	

*Serpine1*	FWD: TTCAGCCCTTGCTTGCCTC	60
	REV: ACACTTTACTCCGAAGTCGGT	

*Serpinh1*	FWD: GGGAACGGATCGCTCCAAA	67
	REV: GGACCTGTGAGGGTTTACCAG	

*Timp1*	FWD: CACGGGCCGCCTAAGGAACG	60
	REV: GGTCATCGGGCCCCAAGGGA	

*Timp2*	FWD: GCCAAAGCAGTGAGCGAGAAG	56
	REV: CACACTGCTGAAGAGGGGGC	

*Timp3*	FWD: AAGAAAAGAGCGGCAGTCCC	60
	REV: TTTGGCCCGGATCACGATG	

*Timp4*	FWD: TATGGTAGGTGGGCTGACTGT	64
	REV: AGTTGAGACAGTGGGAGTAGGA	
